# Cultural competence in mental health nursing: validity and internal consistency of the Portuguese version of the multicultural mental health awareness scale—MMHAS

**DOI:** 10.1186/s12888-016-0848-z

**Published:** 2016-05-17

**Authors:** Ana Paula Teixeira de Almeida Vieira Monteiro, Alexandre Bastos Fernandes

**Affiliations:** Coimbra Nursing School, Rua 5 de Outubro-Apartado 55, 3001-901 Coimbra, Portugal; Centro Hospitalar e Universitário de Coimbra (CHUC), Praceta Prof. Mota Pinto, 3000-075 Coimbra, Portugal

**Keywords:** Mental Health Nurses, Multicultural awareness, Multicultural nursing, Cultural competence, Psychometric testing, Instrument validity, Instrument reliability

## Abstract

**Background:**

Cultural competence is an essential component in rendering effective and culturally responsive services to culturally and ethnically diverse clients. Still, great difficulty exists in assessing the cultural competence of mental health nurses. There are no Portuguese validated measurement instruments to assess cultural competence in mental health nurses. This paper reports a study testing the reliability and validity of the Portuguese version of the Multicultural Mental Health Awareness Scale–MMHAS in a sample of Portuguese nurses.

**Methods:**

Following a standard forward/backward translation into Portuguese, the adapted version of MMHAS, along with a sociodemographic questionnaire, were applied to a sample of 306 Portuguese nurses (299 males, 77 females; ages 21–68 years, *M* = 35.43, *SD* = 9.85 years). A psychometric research design was used with content and construct validity and reliability. Reliability was assessed using internal consistency and item–total correlations. Construct validity was determined using factor analysis.

**Results:**

The factor analysis confirmed that the Portuguese version of MMHAS has a three-factor structure of multicultural competencies (*Awareness, Knowledge*, and *Skills*) explaining 59.51 % of the total variance. Strong content validity and reliability correlations were demonstrated. The Portuguese version of MMHAS has a strong internal consistency, with a Cronbach's alpha of 0.958 for the total scale.

**Conclusions:**

The results supported the construct validity and reliability of the Portuguese version of MMHAS, proving that is a reliable and valid measure of multicultural counselling competencies in mental health nursing. The MMHAS Portuguese version can be used to evaluate the effectiveness of multicultural competency training programs in Portuguese-speaking mental health nurses. The scale can also be a useful in future studies of multicultural competencies in Portuguese-speaking nurses.

## Background

The role of migration in European population change is being debated during recent years as a result of growing concerns about issues such as demographic ageing, shortages of working age populations and payment of pensions among others [1]. In 2015, Europe is suffering from the worst refugee crises since World War II. These refugees are fleeing war, violence and persecution in their country of origin.

Immigrants and asylum-seekers have a higher risk of mental illness due to the fact that they often have been exposed to extreme conditions—for instance, forced migration, significant personal losses and other human rights violations-simply due to the fact that they are refugees in a foreign country, with different cultural norms [2].

Mental health issues including major depression disorder, post-traumatic stress disorder, and general anxiety disorder are common among newly arrived immigrants and refugees. While many immigrants and refugees are resilient, traumatic experiences and migration stressors have a great impact on their mental well-being [3–5].

As the largest group of health professionals in the world, nurses are the key to providing cost effective mental health care to this vulnerable populations, meeting the immigrants and refugees’ complex mental health needs. Nurses are key to healthcare for refugees and migrants [6]. However, most nurses lack competence and confidence in dealing with the distress and mental health problems of refugees and migrants. Lack of cultural competence in mental health among nurses limits the potential to provide high-quality care for the growing number of people with diverse backgrounds [7]. An additional factor that can be a conundrum when examining standards for culturally competent mental health care is the lack of uniformity in educating mental health providers about cultural competence and inconsistencies in how cultural competence is defined [8].

Cultural competence can be seen as a necessary set of skills for mental health nurses to attain in order to render effective patient-centred care. Still, there is limited literature available identifying and describing the instruments that measure mental cultural competence in nursing professionals. These instruments are crucial to assess their cross-cultural strengths and weaknesses in order to design specific training activities or interventions that promote multicultural competence in mental health care [9].

Portuguese is the world’s sixth most spoken language (in America, Africa and Europe there are over 230 million Portuguese speakers) and millions of nurses are using Portuguese as the first language in health care. In spite of this fact, there are no Portuguese validated measurement instruments to assess cultural competence in mental health among nurses.

Cultural competency has been defined as a set of congruent behaviors, attitudes and policies that come together in a system, agency or among professionals that enable that system, agency or those professions to work effectively in cross-cultural situations. It has been seen as the ongoing process in which the health care provider continuously strives to work effectively within the cultural context of the client [10]. Thus, cultural competence is active, developmental, and is aspirational rather than achieved [11].

The tripartite cultural competence model [12, 13] has three components: 1-*Cultural awareness, 2-Cultural knowledge,* and 3-*Cultural skills*. First, the *awareness* component refers to the mental health professional’s awareness of one’s own worldview and cultural biases. *Cultural awareness* is the self-examination and in-depth exploration of one’s own cultural and professional background. In other words, this process involves the recognition of one’s biases, prejudices, and assumptions about individuals who are different. A culturally competent professional is one who is actively in the process of becoming aware of his or her own assumptions about human behavior, values, biases, preconceived notions, and personal limitations. Second, *cultural knowledge* is the process of seeking and obtaining a sound educational foundation about diverse cultural and ethnic groups. In order to obtaining this knowledge base, the health care provider must focus on the integration of three specific issues: health-related beliefs and cultural values, disease incidence and prevalence, and treatment efficacy [14]. A *Multicultural knowledge* requires mental health givers to be knowledgeable about various cultural factors that might influence the therapeutic process. A culturally competent professional is one who actively attempts to understand the worldview of culturally diverse populations—values, assumptions, practices, communication styles, group norms, biases, and personal experiences.

Multicultural competence of mental health practitioners includes the specific skill of adapting treatments and practices to better match the particular needs of the client [15].

The ethnic composition Portuguese population is becoming increasingly diverse in the last two decades. The Portuguese mental health care system is struggling to address some of the specific challenges presented by recent immigration from Africa, Brazil, Eastern Europe and China [16]. For mental health care providers and specifically nurses, the need to provide culturally appropriate and competent care is recognized as essential. Still, little attention has been given to the development of multicultural competencies among Portuguese health care professionals in mental health settings [17,18]. A study with 22 Portuguese nurses, aiming to analyse personal experiences and significant situations of nursing care in multicultural contexts, highlights key multicultural issues that are important for mental health and psychiatric nursing care: therapeutic environment management, roles, speech patterns, and therapeutic communication with minority clients and families [19]. Currently, the instruments to assess cultural competence in nurses and nursing students are self-administered and based on individuals’ own perceptions. The instruments are commonly utilized to test the effectiveness of educational programs designed to increase cultural competence [20]. Therefore, instruments that accurately and reliably measure the knowledge, attitudes, and skills reflecting cultural competence in Portuguese mental health nurses are scarce.

A study was developed to evaluate the cultural diversity competence of Portuguese child care workers (N = 51) [21]. The variables integrated a self-report measure- a cultural competence questionnaire, and an objective measure for evaluation of cultural diversity competencies. But this self-report measure had some limitations, namely the small size of the sample in the validation study, the low Alpha Cronbach’s in two subscales and the fact that it was targeted to child care workers.

## Methods

### Aim

The aim of this study was to translate and test the psychometric properties of the Portuguese version of the Multicultural Mental Health Awareness Scale—MMHAS.

### Design

A psychometric research design was used with content and construct validity and reliability. Reliability was determined with Cronbach’s alpha coefficient.

### Sample/participants

A convenience sample of Registered Nurses (RN) was recruited online using the *LimeService/ LimeSurvey* hosting platform. The research questionnaire was made available online, and participants were invited to participate by email, social networks, and trough professional contacts. The survey took approximately 20 to 30 min to complete and was accessed with a link to *LimeService/ LimeSurvey* hosting platform. The sample is composed of 306 subjects - aged between 21 and 68 years (with an average age of 35.4 years and a standard deviation of 9.8 years); 25.2 % female and 74.8 % male, who agreed to participate in the study and matched the following inclusion criteria: to be a RN in Portugal and proficiency in Portuguese Language.

### Data collection

Data were collected using the Portuguese version of the *Multicultural Mental Health Awareness Scale*–MMHAS, along with a socio-demographic questionnaire.

### Multicultural mental health awareness scale–MMHAS

The original Multicultural Mental Health Awareness Scale (MMHAS) was designed to provide a psychometrically sound instrument to successfully assess professional multicultural competence in mental health and the effectiveness of a multicultural training program in mental health. Items on the new scale were generated in order parallel the Queensland Transcultural Mental Health Centre (QTMHC) training program’s objectives. Results indicated a scale with 35 items and three factors with moderate to high factor loadings and satisfactory psychometric properties. The three factors found to be interrelated were: *Multicultural Counselling Awareness, Multicultural Counselling Knowledge, and Multicultural Counselling Skills,* in line with the multicultural counselling competencies. The Cronbach’s alpha for the total scale was .91. The internal consistency of the *Multicultural Counselling Awareness* subscales was .89, .92 for the *Multicultural Counselling Knowledge* subscale, and .90 for the *Skills* subscale. The MMHAS is unique as it not only reflects language issues and correct intervention strategies, but also assesses the ability to address service barriers and to work with interpreters [22].

### Procedures

The questionnaire was translated using the back translation system by two bilingual translators. After the translations process, a committee including one expert in mental health, one expert in English Language and Literature, also bilingual and, the researcher discussed discrepancies and agreed upon an integrated version of the translation. A group of nursing students reviewed the questionnaire with the investigator to check the appropriateness of the translations and resolved remaining discrepancies in translations. Consensus in terms of semantic, idiomatic, experiential, and conceptual equivalence was reached and a final version of the MMHAS Portuguese version was provided. The study sample was identified using a convenience sampling method. After a process of translation and transcultural validation, the Portuguese version of MMHAS, along with a socio-demographic questionnaire, were applied to a sample of 306 Portuguese nurses. The research questionnaire was made available online, and participants were invited to participate by email, social networks, and trough professional contacts. The information of the study was disseminated online in several nursing professional networks and social network sites (Facebook, Portuguese Nursing Blogs; Portuguese Nursing sites). Only a few participants were directed notified, for ex. with a personnel email with a link.

The survey took approximately 20 to 30 min to complete and was accessed with a link to *LimeService/ LimeSurvey* hosting platform. *LimeSurvey* provides a web-based product for internet surveys with more than twenty different question formats, in addition to survey tokens that prevent repeat participants, and branching surveys that offer different questions depending on prior responses. Moreover, the survey administrator can control how the survey interface will appear to participants through a built–in editor. Surveys were available online from November 2013 to February 2014. Once the recruitment period had ended all data was condensed into a file, downloaded into a database, exported into SPSS and, statistical analysis was conducted. It was not possible to determinate a response rate.

### Ethical considerations

Internet data collection can raise particular, sometimes non-obvious challenges in adhering to ethical principles. In this study we followed the Ethics Guidelines for Internet Mediated Research [23] and the American Psychological Association's Guidelines [24]. Study inclusion required informed consent. Participants were informed that they could withdraw from the study at any time. Regarding ethical aspects in research, it is worth highlighting that authorization to make cultural adaptation and validation of the MMHA for the Portuguese reality was obtained from the author of the original instrument. The study was formally approved by the Ethical Committee for Health Research of Coimbra Nursing School.

### Data analysis

Data analyses were completed with the use of Statistical Package for the Social Sciences version 20.0 (SPSS, Chicago, IL, USA). To explore the MMHAS internal consistency, Cronbach’s *alpha* was obtained, as well as item-total correlations and alpha values when the item was deleted. Exploratory factor analysis (principal component analysis) was carried out to identify factor structure of MMHAS (Portuguese version).

## Results

### Participants

The sample was composed of 306 subjects—aged between 21 and 68 years, with an average age of 35.43 years and a standard deviation of 9.85 years; 299 males (74.8 %) and 77 females (25.2 %).

Demographic characteristics of the respondents are summarized in Table 1.

**Table 1:** Sociodemographic characterization of the sample

	Number	Percent
Age range		
[20–30[ [30–40[ [40–50[ [50–60[ [60–70]	104 101 72 26 3	34.0 33.0 23.5 8.5 1.0
$\overline{\mathrm{x}}$ = 35.43; Md = 33.00; s = 9.85; x_min_ = 21.00; x_máx_ = 68.00;	*p* = 0.000	
Gender		
Female Male	77 229	25.2 74.8
Marital status		
Single Married Divorced/Separated Widowed Civil Union	133 127 14 3 29	43.5 41.5 4.6 1.0 9.5
Profissional status		
RN Graduated Nurse Specialized Nurse Unemployed	300 1 4 1	98.0 0.3 1.3 0.3
Professional experience (years)		
< 5 [5–10[ [10–15[ [15–20[ [20–25[ [25–30[ [30–35[ ≥ 35	82 67 46 27 37 26 14 4	26.8 21.9 15.0 8.8 12.1 8.5 4.6 1.3
$\overline{\mathrm{x}}$ = 12.23; Md = 10.00; s = 9.79; x_min_ = 0.00; x_máx_ = 42.00; *p* = 0.000		

### Principal component analysis

In our exploratory factor analysis, the Kaiser Mayer Olkin Measure and the Bartlett's chi square tests were checked for the appropriateness of data for factor analysis. Both the adequacy of the sample and the use of factor analysis on the data were confirmed.

The *Kaiser-Meyer-Olkin* (KMO) measure of sampling adequacy was 0.949, exceeding the benchmark value of 0.60. The *Bartlett's Test of Spehericity* (*χ*2 = 7776.696; p = 0.000) was statistically significant, supporting the factorability of the correlation matrix. A principal component analysis (PCA) with orthogonal *varimax rotation* and combined scree plot test and parallel analysis yielded a three factor solution that accounted for 59.4 % of the total variance explained (Table 2). A principal component analysis (PCA) with orthogonal varimax procedure was employed to rotate the factors to a simple structure in order to determine the number of factors to retain. Items with a loading of greater than 0.50 were retained for a specific factor. Split items were retained to factors if the square of the loadings for a factor was >50 % that of its loading on any other factor (Table 2).

**Table 2:** Loadings (item-component correlations) obtained by PCA

Item	Factors		
	C1	C2	C3
1.	0.147	0.508	0.222
2.	0.278	0.602	0.191
3.	0.214	0.703	0.199
4.	0.128	0.810	0.093
5.	0.192	0.749	0.164
6.	0.174	0.792	0.188
7.	0.221	0.626	0.346
8.	0.148	0.782	0.182
9.	0.143	0.810	0.183
10.	0.047	0.788	0.262
11.	0.101	0.625	0.362
12.	0.614	0.246	0.062
13.	0.715	0.224	0.147
14.	0.842	0.152	0.141
15.	0.840	0.130	0.168
16.	0.746	0.111	0.251
17.	0.584	0.300	0.261
18.	0.731	0.136	0.304
19.	0.743	0.095	0.281
20.	0.767	0.137	0.273
21.	0.648	0.154	0.252
22.	0.509	0.283	0.397
23.	0.544	0.260	0.356
24.	0.659	0.090	0.316
25.	0.273	0.050	0.551
26.	0.302	0.089	0.541
27.	0.287	0.222	0.707
28.	0.241	0.221	0.751
29.	0.346	0.271	0.711
30.	0.225	0.281	0.765
31.	0.181	0.349	0.747
32.	0.332	0.213	0.692
33.	0.159	0.263	0.748
34.	0.189	0.276	0.667
35.	0.104	0.289	0.519
Value	14.8	3.7	2.3
Explained variance. (%)	42.3	10.5	6.6

Rotation Method: Varimax with Kaiser Normalization

Extraction Method: Principal Component Analysis

The items of the MMHAS Portuguese version total scale can be divided into three subscales:

- Factor 1-*Cultural Awareness*, with thirteen items (12, 13, 14, 15, 16, 17, 18, 19, 20, 21, 22, 23 and 24).
- Factor 2-*Cultural Knowledge*, with eleven items (1, 2, 3, 4, 5, 6, 7, 8, 9, 10 and 11).
- Factor 3-*Cultural Competence/Skills*, with eleven items (25, 26, 27, 28, 29, 30, 31, 32, 33, 34 and 35).

As can be seen from Table 2, all the items (except item 1 and item 2) load onto their original subscales with loading values of .90 or greater in each case. The Item 1-“*My knowledge of various cultures is”* in the original questionnaire*,* and in the Portuguese version *“o meu conhecimento sobre outras culturas é*”-in the original study of the MMHA loaded in the Factor 1 (Cultural Awareness) and in the Portuguese version loaded in the Factor 2 (Cultural Knowledge). Also the Item 2-“*My knowledge of acculturation is*” in the original questionnaire*,* and in the Portuguese version *“O meu conhecimento sobre o processo de aculturação é*”-in the original study of the MMHA loaded in the Factor 1 (Cultural Awareness) and in the Portuguese version loaded in the Factor 2 (Cultural Knowledge). The variations can be explained by some cultural, semantic and linguistic differences between the Portuguese and English versions. The mean inter-item correlations, which can be regarded as an indicator of the homogeneity of the scale, were also computed. In the sample, the mean inter item correlations are rather high.

### Internal consistency reliability

The overall reliability coefficient Cronbach’s alpha, is based on the average correlation of items within the scale. Cronbach’s alpha coefficient of reliability of the MMHAS Portuguese version is 0.958. For the subscale *Multicultural Awareness* Cronbach’s alpha is 0.938; for the subscale *Multicultural Knowledge* Cronbach’s alpha is 0.927 and, for the subscale *Multicultural Competence/Skills* Cronbach’s alpha is 0.922 (Table 3).

**Table 3:** Reliability Statistics-Cronbach’s α coefficients of reliability

Item	Awareness		Knowledge		Skills		Total MMHAS	
	A	B	A	B	A	B	A	B
01	–	–	0.524	0.928	–	–	0.459	0.958
02	–	–	0.630	0.924	–	–	0.572	0.957
03	–	–	0.705	0.921	–	–	0.587	0.957
04	–	–	0.756	0.918	–	–	0.528	0.958
05	–	–	0.727	0.920	–	–	0.578	0.957
06	–	–	0.773	0.918	–	–	0.603	0.957
07	–	–	0.673	0.923	–	–	0.639	0.957
08	–	–	0.760	0.918	–	–	0.579	0.957
09	–	–	0.785	0.917	–	–	0.589	0.957
10	–	–	0.767	0.918	–	–	0.564	0.957
11	–	–	0.650	0.923	–	–	0.570	0.957
12	0.571	0.938	–	–	–	–	0.520	0.958
13	0.704	0.934	–	–	–	–	0.627	0.957
14	0.799	0.931	–	–	–	–	0.665	0.957
15	0.798	0.931	–	–	–	–	0.667	0.957
16	0.737	0.933	–	–	–	–	0.645	0.957
17	0.652	0.936	–	–	–	–	0.649	0.957
18	0.757	0.932	–	–	–	–	0.679	0.957
19	0.746	0.932	–	–	–	–	0.650	0.957
20	0.778	0.932	–	–	–	–	0.688	0.957
21	0.663	0.935	–	–	–	–	0.605	0.957
22	0.677	0.935	–	–	–	–	0.731	0.956
23	0.693	0.934	–	–	–	–	0.717	0.956
24	0.688	0.934	–	–	–	–	0.614	0.957
25	–	–	–	–	0.546	0.922	0.493	0.958
26	–	–	–	–	0.577	0.920	0.526	0.958
27	–	–	–	–	0.728	0.913	0.677	0.957
28	–	–	–	–	0.767	0.912	0.672	0.957
29	–	–	–	–	0.789	0.911	0.744	0.956
30	–	–	–	–	0.779	0.911	0.701	0.957
31	–	–	–	–	0.768	0.912	0.698	0.957
32	–	–	–	–	0.740	0.913	0.692	0.957
33	–	–	–	–	0.734	0.913	0.641	0.957
34	–	–	–	–	0.691	0.915	0.622	0.957
35	–	–	–	–	0.523	0.925	0.491	0.958
Cronbach’s Alpha	0.938		0.927		0.922		0.958	

A-Item-total correlation; B-Cronbach’s Alpha if item Deleted

The extent to which individual items affect the reliability of the scale can be examined by calculating Cronbach’s alpha when each item is removed from the scale. The removal of any single item would not significantly increase the reliability of the scale: in all the items of the scale where the alpha if deleted isn’t higher than the overall alpha and in subscales (Table 3).

## Discussion

Cultural competence is an essential component in rendering effective and culturally responsive services to culturally and ethnically diverse clients [25]. Because providing culturally competent care is essential in nursing, the measurement of cultural competence and its effect on patient outcomes is central to the discipline. Despite the limitations associated with existing instruments, there is much value in the initial assessment of cultural competence they provide as well as tracking measurements of cultural competence over time. The aim of the study was to investigate whether the Portuguese version of the *MMHAS* has satisfactory psychometric properties. The procedures of translation and cultural adaptation represented no major problems and gave rise to a reliable Portuguese version of the MMHAS. PCA was carried out in order to re-examine the factor structure of the scale. The three-factor solution accounted for 54.9 % of the total variance. The items of the MMHAS Portuguese version total scale can be divided into three subscales: *Cultural Awareness*, *Cultural Knowledge*, and *Cultural Competence/Skills*, according the tripartite cultural competence model [26]. Also some transcultural nursing models underlies the cultural awareness, cultural knowledge, and cultural skills as crucial components in the process of cultural competence in the delivery of healthcare services.

Excellent Cronbach’s *alpha* coefficients for the three subscales and total scale, and satisfactory item-total coefficients for the correspondent items confirmed that the MMHAS subscales are internally consistent, with the correspondent items properly correlated with each other [27–29]. The results for internal consistency were similar to those obtained by in the original study.

In this study, 74.8 % of the respondents were male, which does not match the sociodemographic composition of Portuguese professional nurses. Some emerging literature points out the correlation between demographic characteristics of subjects and online survey response behavior by investigating how socio-demographic factors, gender in particular, affect online survey response behavior. This is because differences in the way females and males inhabit cyberspace may exaggerate the effects of differences in how females and males undergo social exchange, resulting in differences in online survey response rates [30]. Another explanation is that some of the participants were psychiatric/mental health nurse practitioners, with a considerable number of male nurses.

The MMHAS displayed an apropriate internal consistency. The findings show that the Portuguese version of the MMHAS adequately addressed the original concepts and dimensions, and demonstrated a high level of equivalence with the original version. The findings indicate a remarkable consistency in the factor structure of the Portuguese version. The concepts embedded in this cultural competence questionnaire, provide an ideal structure for educational programs. The Portuguese version of the MMHAS can be used to evaluate the effectiveness of multicultural competency training programs in Portuguese-speaking mental health nurses. The scale can also be a useful in future studies of multicultural competencies in Portuguese nurses.

### Limitations

This study has some limitations including the sample representativeness (74.8 % male), which does not match the sociodemographic composition of Portuguese professional nurses. Another limitation of this study was the use of a convenience sample, with the inherent bias. Future studies should have larger sample sizes and include gender representative samples, and look at the psychometric performance of the MMHA.

## Conclusion

The results of this study support the construct validity and reliability of the Portuguese version of MMHAS, proving that is a reliable and valid measure of multicultural counselling competencies in mental health. This study suggests that MMHAS could be an important addition to the compendium of instruments used to assess Multicultural Mental Health Competences in Nurses, and also contributes to improve culturally sensitive nursing care.

The MMHAS Portuguese version can be used to evaluate the effectiveness of multicultural competency training programs in Portuguese-speaking mental health nurses. The scale can also be useful in future studies to access multicultural competencies in Portuguese-speaking nurses all around the world.

## Ethics and consent to participate

Study inclusion required informed consent. Participants were informed that they could withdraw from the study at any time. The study was formally approved by the Ethical Committee for Health Research of Coimbra Nursing School-*Comissão de Ética da Unidade de Investigação em Ciências da Saúde: Enfermagem da Escola Superior de Enfermagem de Coimbra*, Portugal, Committee’s reference number:176–072013.

## Consent to publish

Not applicable.

## Availability of data and materials

All the data supporting these findings is contained within the manuscript.

## Abbreviations

MMHAS: Multicultural Mental Health Awareness Scale
PCA: Principal components analysis
QTMHC: Queensland Transcultural Mental Health Centre
RN: Registered Nurses
SPSS: Statistical Package for the Social Sciences
